# Realized Volatility and Absolute Return Volatility: A Comparison Indicating Market Risk

**DOI:** 10.1371/journal.pone.0102940

**Published:** 2014-07-23

**Authors:** Zeyu Zheng, Zhi Qiao, Tetsuya Takaishi, H. Eugene Stanley, Baowen Li

**Affiliations:** 1 Shenyang Institute of Automation, Chinese Academy of Sciences, Shenyang, P.R. China; 2 Department of Physics and Centre for Computational Science and Engineering, National University of Singapore, Singapore, Republic of Singapore; 3 NUS Graduate School for Integrative Sciences and Engineering, National University of Singapore, Singapore, Republic of Singapore; 4 Hiroshima University of Economics, Hiroshima, Japan; 5 Center for Polymer Studies and Department of Physics, Boston University, Boston, Massachusetts, United States of America; University of Maribor, Slovenia

## Abstract

Measuring volatility in financial markets is a primary challenge in the theory and practice of risk management and is essential when developing investment strategies. Although the vast literature on the topic describes many different models, two nonparametric measurements have emerged and received wide use over the past decade: realized volatility and absolute return volatility. The former is strongly favored in the financial sector and the latter by econophysicists. We examine the memory and clustering features of these two methods and find that both enable strong predictions. We compare the two in detail and find that although realized volatility has a better short-term effect that allows predictions of near-future market behavior, absolute return volatility is easier to calculate and, as a risk indicator, has approximately the same sensitivity as realized volatility. Our detailed empirical analysis yields valuable guidelines for both researchers and market participants because it provides a significantly clearer comparison of the strengths and weaknesses of the two methods.

## Introduction

In recent decades, financial markets have grown rapidly and financial instruments have become increasingly complex. The result is a market that is highly volatile and that produces a level of risk that strongly affects all investment decisions [1]. The ever-growing need for theoretical and empirical risk indicators has driven a rapid expansion of research on price volatility in financial markets. Since volatility is strongly linked to uncertainty, it is a key input in many investment decisions and in overall portfolio management. Because investors and portfolio managers must determine what levels of risk they can bear and because volatility is the primary risk indicator [2], reliable forecasts of market volatility are pivotal. Thus comparing the predictive capabilities of existing methods of quantifying market volatility can potentially produce extremely valuable information for both market researchers and active traders.

Financial market volatility is a quantity that is difficult to observe. Although we can watch instrument prices and their movement on a monitor, we cannot directly "watch" volatility. Volatility must be approximated using calculations that draw on such observable values as daily price changes or intraday price changes, and these volatility calculation techniques fall into roughly two categories: parametric methods and nonparametric methods [3].

Parametric approaches to volatility modeling are based on explicit functional form assumptions regarding the volatility and include both discrete-time models and continuous-time models. The most widely used discrete-time models are the ARCH model [4] and stochastic volatility (SV) model. Much has been written about the ARCH model and it has been modified into dozens of different variations, e.g., the generalized autoregressive conditional heteroskedasticity model (GARCH) [5]. In parallel with the ARCH class of models, SV models are based on an autoregressive formulation of a continuous function describing the latent volatility process [6]. In contrast to discrete-time models, most continuous-time models are used in the development of asset and derivative pricing theories. They assume that the sample paths are continuous, and they model the corresponding diffusion processes in the form of stochastic differential equations [7].

In recent years these parametric models have become increasingly restrictive and difficult to use, and there has been an movement toward the use of flexible and computationally simple nonparametric measurements, two of which are widely used: absolute return volatility and realized volatility.

The simplest measurement of instrument price volatility is tracking the absolute return values and observing the range of day-to-day price changes. This traditional method of volatility modeling from daily returns measures the log-difference of closing prices. Treating absolute returns as a proxy for volatility is the basis of much of the modeling efforts presented in the literature [8]–[10]. It has been used primarily in econometrics and econophysics research [11]–[14] and, in recent years, has shown itself to be a better measurement of volatility [15].

The second method, measuring realized volatility, summarizes all the variances sampled at regular intra-daily intervals under some assumptions of the quadratic variation of the underlying diffusion process [16]–[18]. Realized volatility measurements, which track the variance of price changes on an intra-day basis, have become possible in recent years because of the increasing availability of high frequency data. Although this volatility measurement derived from high frequency data is more accurate and in principle a better aid in forecasting volatility, it exhibits numerous micro-structural problems. Price discreteness, bid-ask bounce [19], screen fighting [20], non-trading hours, and the irregular spacing of quotes and transactions can all bias volatility estimates. By appropriately adjusting bias and investigating returns standardized by realized volatility, it is found that the return dynamics are consistent with a Gaussian stochastic process incorporating time-varying volatility [21]–[24].

In this paper we compare the two most popular nonparametric volatilities—absolute return volatility and realized volatility—and focus on their accuracy as risk indicators, their short-term effect, and their long-term memory. Because realized volatility reflects intra-day variance and absolute return volatility reflects day-to-day change, we will also determine ways in which they differ. Our comparison will provide a clear understanding of the advantages and disadvantages of these two measurements, and this will make possible the development of better guidelines for both researchers and market participants.

## Results

**Figure 1:**
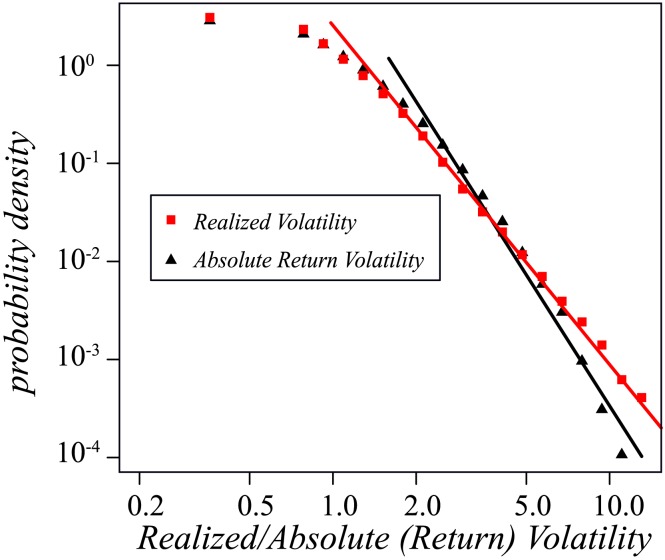
The probability density function of absolute return volatility and realized volatility of TOPIX Core30 Index members drawn on a log-log plot. Both of them follow power-law distribution. The slope of realized volatility is 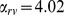 a bit larger than that of absolute return volatility 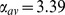, which indicates that realized volatility has slightly larger fat tails than absolute return volatility. For realized volatility about 1996 of the 2500 power law fitness KS tests fail to reject the null while for absolute return volatility about 1482 of the 2500 power law fitness KS tests failed to reject the null. The results suggest that the power law distribution may fit both of them but realized volatility has better fit with power law compared to absolute return volatility. The power law fitness KS test details may refer [30], [31].

Figure 1 shows a log-log plot of the probability density function for (a) the absolute return volatility and (b) the realized volatility. Notice that both become a straight line in the tails, indicating that both volatilities follow a power-law distribution. The fat tails indicate that the probability that the absolute return volatility or realized volatility will be significantly large is higher than would be indicated by a Gaussian (normal) distribution. The tails of the realized volatility are somewhat fatter than the tails of the absolute return volatility, indicating that its fluctuations are stronger. This is because the absolute return volatility captures only the change in daily closing price, while the realized volatility captures data on the basis of quotes sampled at discrete intervals throughout the day. Note that using these two volatility calculation methods means that a zero return will not provide useful information for a given trading day. It also means that although a high return may signal a high absolute return volatility during the day, it may also simply indicate that the opening price is significantly different from the closing price the previous day but very close to the closing price of the same trading day, and have a small high-low spread. On the other hand, realized volatility can capture this phenomenon exactly and thus will offer more insights into price-change behavior.

**Figure 2:**
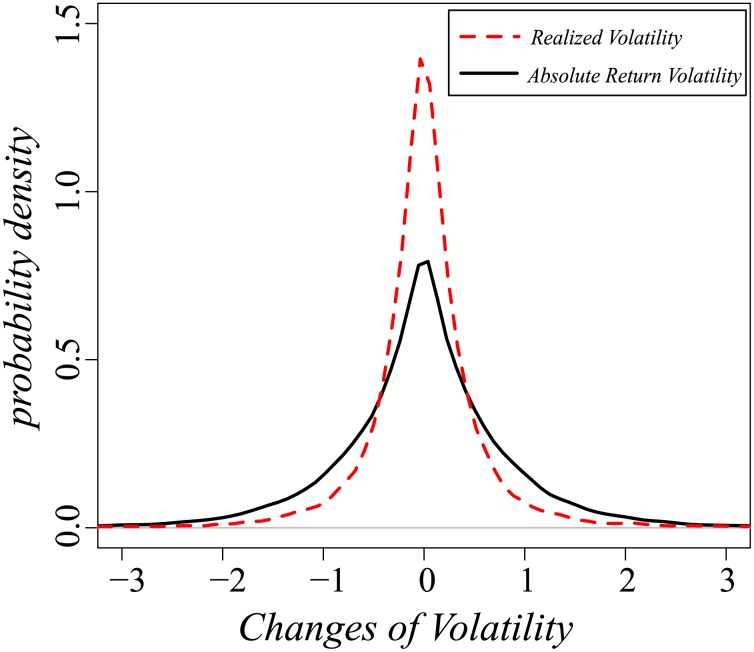
The distribution peak (near 0) of realized volatility changes between neighboring days 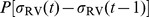 is much sharper than of absolute return volatility changes 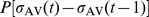. The kurtosis of realized volatility is 105 which is much higher than the kurtosis of absolute return volatility which is 61. Furthermore since we had normalized the variance of both values to 1. The differ of kurtosis are mostly contributed by the relations between neighboring days. The result indicates that the realized volatility is much smoother than absolute return volatility. Black curve stands for absolute return volatility of 30 TOPIX Core30 Index members while red dash curve represents realized volatility.

We next examine the ways in which the two methods of calculating volatility differ and draw a distribution of the daily changes in both. Figure 2 shows that the probability density of the daily change of realized volatility (red dashes) is sharper than that of absolute return volatility (black line) and that both distributions exhibit positive excess kurtosis, i.e., they are leptokurtic. The kurtosis of the daily changes for realized volatility is larger, indicting that it is more "stable" than absolute volatility and that there is a smaller probability it will exhibit large fluctuations. In other words, realized volatility can usefully model the clustering properties of volatility in which random periods of low activity are followed by periods of high activity, a behavior often observed in financial markets.

Note that both methods of calculating volatility allow us to calculate and analyze fat-tail and clustering properties. In order to understand the underlying dynamics of these two features, we study the memory effect in both methods.

**Figure 3:**
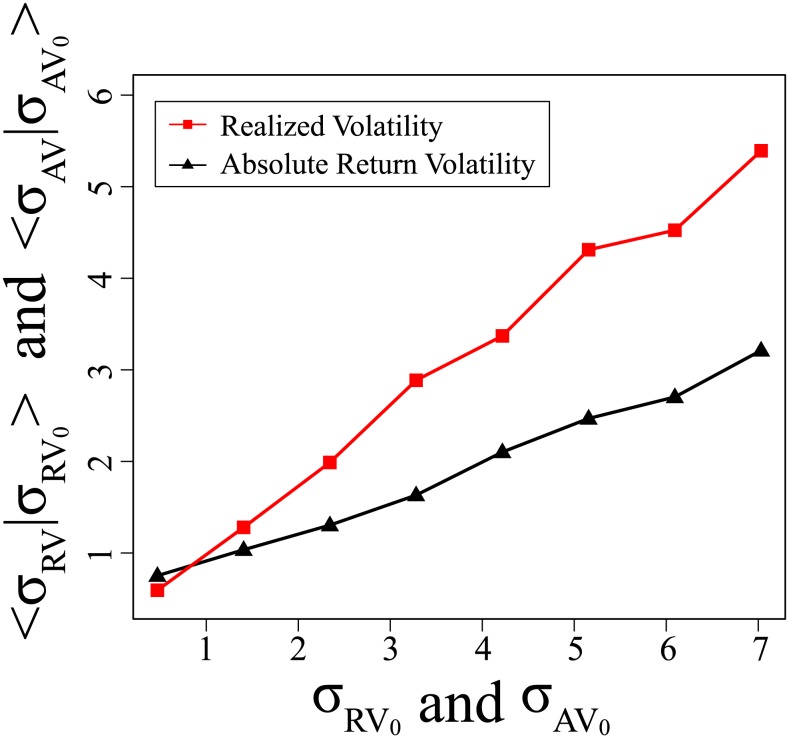
Short-term effect of realized volatility is stronger than that of absolute return volatility. Shown is the mean conditional volatility 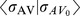 and 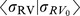 for both absolute return volatility (black triangles) and realized volatility (red squares). Compared to absolute return volatility, realized volatility has stronger short-term effect because the red square line is above the black triangle line all the time except for the lower left points.

We begin by examining the short-term memory effect. Figure 3 shows the mean conditional volatility for both absolute return volatility and realized volatility, which is the first moment of 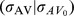 and 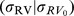, immediately after a given 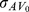 or 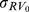 subset. Note that both the absolute return volatility and the realized volatility have a short-term effect, i.e., the large 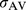 or 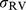 tend to follow large 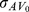 or 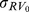 and the small 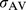 or 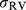 tend to follow small 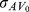 or 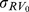. The realized volatility has a stronger short-term effect than the absolute return volatility, however. The line connecting the red squares (the mean conditional realized volatility) remains above the line connecting the black triangles (mean conditional absolute return volatility) at all points except at the lower left.

**Figure 4:**
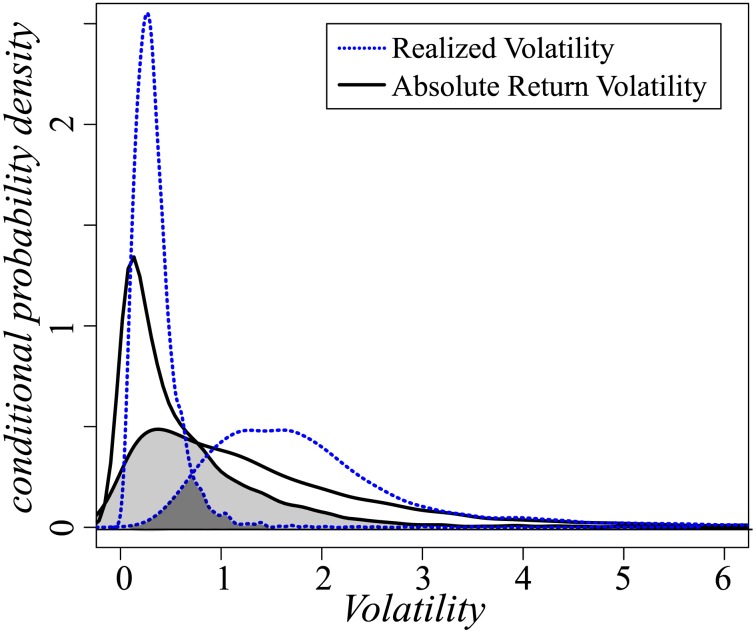
The conditional probability density for the largest and smallest 1/6th portion of the absolute return volatility (black line) and realized volatility (blue dots). The cross-over area (gray area) of absolute return volatility is much larger than the cross-over area (dark gray area) of realized volatility. Noted that we had normalized the variance of both values to 1, the results may mostly reflect that the neighboring days' memory of 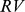 and 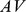 are significantly different.

Figure 4 shows the probability density function of the mean conditional absolute return volatility and the realized volatility given the smallest 1/6th and the largest 1/6th of the whole value. The plot shows that the two lines indicating the smallest and the largest 1/6th portions have a repeated area, which is highlighted in gray. The repeated area (gray area) of the absolute return volatility is much larger than the repeated area (deep gray area) of the realized volatility, indicating that the fluctuations of the realized volatility are much smaller and thus easier to predict over the short term. This supports what is shown in Fig. 3, i.e., that realized volatility better demonstrates the short-term effect, and supports the "clustering feathers" pattern shown in Fig. 2.

**Figure 5:**
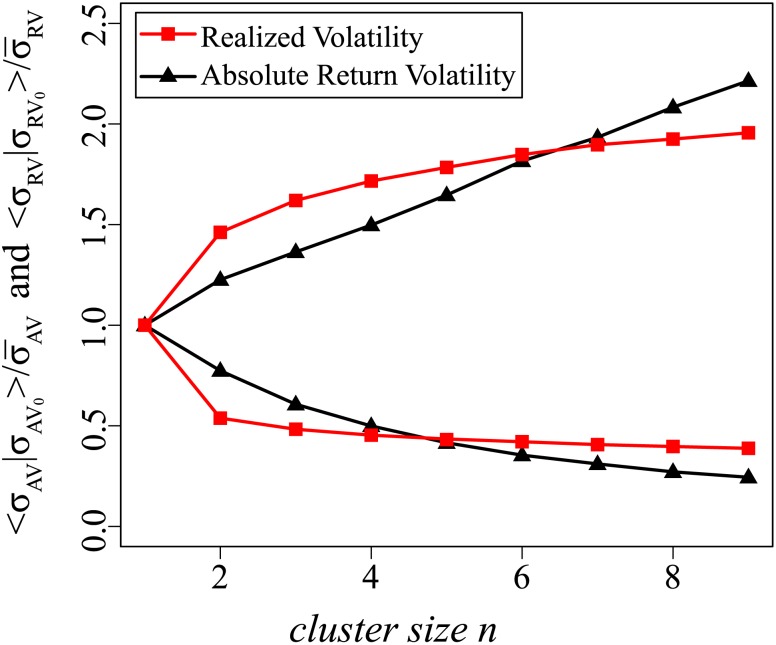
Long term memory effect in volatility subset clusters. Shown is the mean conditional volatility of the absolute return volatility (black triangles) and the realized volatility (red squares) given 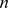 consecutive values that are above (+) or below (−) the median of the entire volatility data set. The upper part of the curves is for + clusters while the lower part is for – clusters. For the + clusters, the mean conditional volatilities for both methods increase with the size of the cluster, behavior opposite to that for the – clusters, indicating the presence of long-term memory in both volatility methods.

The quantities 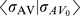 and 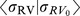 and the smallest and the largest portions of the probability density function accurately describe the short-term memory in both methods. The long-term memory effect in the two volatility methods is equally important. Figure 5 shows the mean conditional volatility of a cluster of 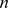 volatility subsets through the dataset. To obtain good statistics we divide the sequence into two bins separated by the median of the entire database. We indicate subsets above the median with "+" and below with "–." Thus 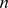 consecutive "+" or "–" subsets form a cluster. The mean of the conditional volatility of an 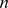-cluster reveals the memory range in the sequence. Figure 5 shows that for "+" clusters the mean conditional volatilities in both methods increase with the size of the cluster. The opposite is true for the "–" clusters. Because we do not see a plateau of large clusters in either method, the results indicate that there is long-term memory in both methods. Note that when we compare these two curves we find that for small intervals the realized volatility (the line connecting the red squares) has a stronger memory effect because it expands more than the absolute return volatility (the line connecting black triangles), which is in accord with the short-term memory behavior shown in Figs. 3 and 4. For longer intervals, however, the slope of the absolute return volatility is larger than the realized volatility, which indicates a stronger long-term memory effect.

**Figure 6:**
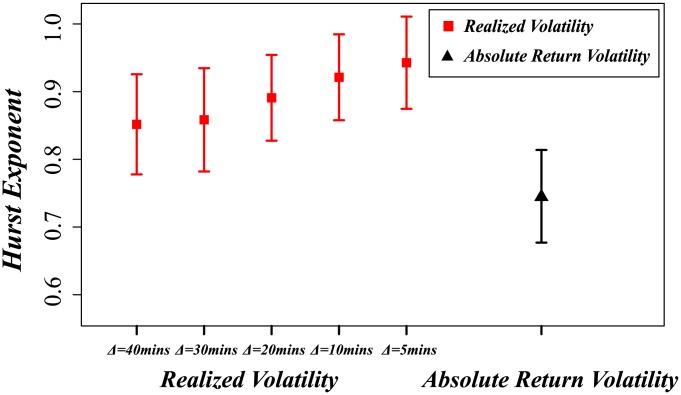
Hurst exponents of realized volatility (squares) are significant higher than the hurst exponent of absolute return volatility (triangles). Additionally the Hurst exponent of realized volatility increases with the decreasing of sampling interval 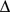.

To confirm the above long-term memory effect picture, we study the Hurst exponent for both methods. The Hurst exponent measures the long-term memory of a time series in terms of the autocorrelations in the time series and the rate at which they decrease as the lag between pairs of values increases. Designated the "index of dependence" or "index of long-range dependence," the Hurst exponent is an widely-accepted method of quantifying the tendency of a time series to either regress strongly to the mean or to cluster in a single direction [25]. A value 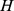 in the range 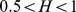 indicates that the time series has long-term positive autocorrelation, i.e., that a high value in the series will probably be followed by another high value and that the future long-term values will also be high. Figure 6 shows the Hurst exponent for both absolute return volatility and realized volatility. Both Hurst exponents are in the range of 0.5 to 1, which means that both methods have a strong autocorrelation with long-term memory effects, i.e., the same result as shown in Fig. 5. The Hurst exponents of realized volatility also increase as sampling interval 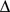 decreases, but all of the values are significantly higher than those of the absolute return volatility.

**Figure 7:**
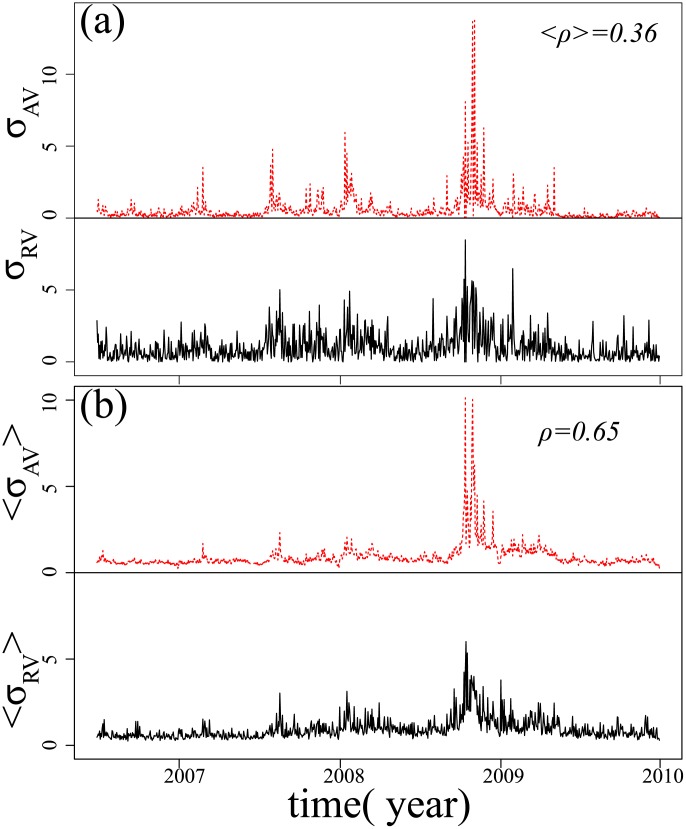
The cross correlation between average realized volatility and average absolute return volatility is much higher than cross correlation between any separate realized volatility and absolute return volatility of each stock. (a) shows an example time series, realized volatility 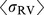 and absolute return volatility 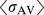 of the stock Nintendo, and the average correlation coefficients of all TOPIX Core30 components 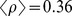; (b) shows the average 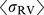 and 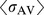 time series of all TOPIX Core30 components with the correlation coefficient between them is 0.65.

Because absolute return volatility and realized volatility are two of the most widely used calculation methods for determining market price fluctuations, they should exhibit strong cross correlations. Surprisingly, when we draw the two time series 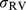 and 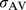 for each stock, we find that the cross correlation values between the two time series are not high, although they appear similar, e.g., the Nintendo stock in Fig. 7(a). We also find that the correlation coefficients of these two quantities for each stock are very low and that the average correlation coefficient for the TOPIX Core30 component 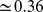. Figure 7(b) shows the time series of the average realized volatility 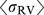 and average absolute return volatility 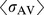 of all TOPIX Core30 components. Surprisingly, we find that the correlation coefficient between 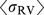 and 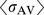 is 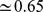, which is much larger than the average correlation coefficients of the two quantities of each separate stock. This correlation coefficient is also larger than any of the correlation coefficients of the two quantities of each stock, the largest of which is 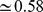.

**Figure 8:**
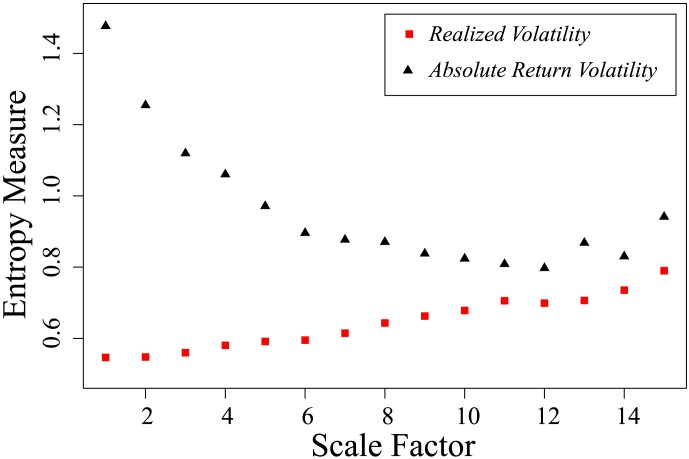
Different multiscale entropy patterns for average realized volatility 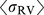 (squares) and average absolute return volatility 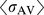 (triangles). The values of entropy depend on the scale factor. For scale one, 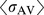 time series are assigned the much higher value of entropy than the entropy value for 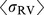 time series. Following the increase of the scale, the value of entropy for 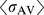 decrease, while the entropy value for 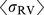 is increasing. Two entropy values become closer for lager scales.

Applying multiscale entropy (MSE) analysis [26] to the two average volatility time series, 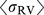 and 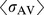 (see Fig. 8). The method of multiscale entropy (MSE) analysis is useful for investigating complexity in time series that have correlations at multiple scale. MSE has been widely applied to a wide variety of time series data to analyze the complexity and memory effect. Figure 8 shows that at scale one the entropy for 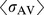 is much higher than entropy for 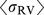. Furthermore, the value of entropy derived from 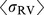 increases with the scale factor, while the value of entropy derived from 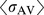 decreases with the scale factor.

## Discussion

In this paper we use several methods to study the clustering and memory effects in two commonly used nonparametric methods of calculating volatility, absolute return volatility and realized volatility. We apply them to both intraday data and daily data and find that both methods are good indicators of market risk because they clearly show the fat-tail and clustering behavior of market price fluctuations. We analyze the short-term and long-term memory effects generated by both methods and find that both offer good predictions of future market behavior. Realized volatility is a better method for describing short-term effects than absolute return volatility and thus it provides a better estimate of near-future possible risk. When we measure the long-term memory capabilities, the two methods are almost the same. Both are sensitive to financial crises, as is shown in their detection of the 2008 global financial crisis. Our analytic comparison of the two approaches will provide researchers and market traders with a more complete understanding of their choices when using volatility as a risk indicator.

The realized volatility and absolute return volatility can both be considered indicators of risk, and we do not find significant correlations between them, but the correlations between the average realized volatility and the average absolute return volatility are very strong with a correlation coefficient 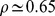, much higher than the correlation coefficient of any individual stock. Our results indicate that the time series of realized volatility and absolute return volatility probably exhibit similar trends. The process of averaging can make the random noise weaker. Additionally, taking into consideration the close relationship between risk and volatility, we may assume that this trend is related to systematic risk.

Finally we use multiscale entropy (MSE) to investigate the averaged realized volatility and absolute return volatility and get somewhat different results. The different entropy changing patterns across different scales clearly indicate that the configurations and behaviors observed when using the realized volatility method differ from those observed when using the absolute return volatility method.

## Materials and Methods

We analyze 30 stocks comprising the TOPIX Core30 Index of the Tokyo Stock Exchange. The time period of the data is from 3 July 2006 to 30 December 2009. Because the calculation methods for realized volatility differ from those of absolute return volatility, we clarify the comparison by using two different representations of volatility. For realized volatility we utilize high-frequency minute-to-minute data and for absolute return volatility we use the daily closing prices.

### Realized volatility

The realized volatility is a model-free estimate of volatility constructed as a sum of squared returns. For high-frequency data, the realized volatility 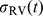 of the 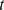 th day is constructed using a sum of 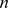 squared intraday returns defined as
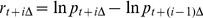
(1)


where, 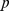 represents the price and 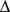 is the sampling interval. Thus the original realized volatility (non-normalized) can be defined
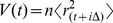
(2)


where 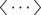 is the daily average value. A good sampling frequency that reduces the bias but maintains the accuracy of the realized volatility measurement is needed if distortion caused by microstructural noise is to be avoided. The long-memory will decrease as 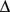 increases, but an extremely short interval 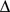 can yield an extremely irregular and unpredictable volatility measurement. We select a sampling frequency of five minutes as possibly yielding the best estimate of the the realized volatility [27]–[29]. The daily realized volatility can then be normalized as
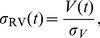
(3)


where 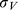 indicates the standard deviation of the original realized volatility series.

### Absolute return volatility

In econophysics research, the daily logarithmic returns are used to calculate the absolute return volatility. For each stock, the daily logarithmic change 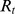 of price 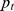, commonly called the return, is
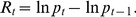
(4)


The daily absolute return volatility is normalized as
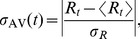
(5)


where 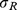 indicates the standard deviation of the return series.

## References

[1] Christoffersen PF, Diebold FX (2000) How relevant is volatility forecasting for financial risk man-agement? Review of Economics and Statistics 82: 12–22.

[2] Green TC, Figlewski S (1999) Market risk and model risk for a financial institution writing options. J Finance 54: 1465.

[3] Andersen T, Bollerslev T, Diebold F (2002) Handbook of Financial Econometrics. Amsterdam: North Holland, Amsterdam.

[4] Engle R (1982) Autoregressive conditional heteroscedasticity with estimates of the variance of united kingdom ination. Econometrica 50: 987.

[5] Bollerslev T (1986) Generalized autoregressive conditional heteroskedasticity. Journal of Econometrics 31: 307.

[6] Taylor S (1994) Modeling stochastic volatility: A review and comparative study. Mathematical Finance 4: 183.

[7] Protter P (1992) Stochastic Integration and Differential Equations: A New Approach, 2nd Edition. New York: Springer-Verlag, New York.

[8] Taylor S (1987) Forecasting of the volatility of currency exchange rates. Int J Forecast 3: 159.

[9] Ding Z, Granger C, Engle R (1993) A long memory property of stock market returns and a new model. Empirical Finance 1: 83.

[10] Granger C, Sin C (2000) Modelling the absolute returns of different stock market indices: exploring the forecastability of an alternative measure of risk. J Forecast 19: 277.

[11] Cizeau P, Liu Y, Meyer M, Peng CK, Stanley H (1997) Volatility distribution in the s&p500 stock index. Physica A 245: 441.

[12] Zheng Z, Yamasaki K, Tenenbaum J, Stanley H (2013) Carbon-dioxide emissions trading and hierarchical structure in worldwide finance and commodities markets. Phys Rev E 87: 012814. 10.1103/PhysRevE.87.012814 23410395

[13] Zheng Z, Podobnik B, Feng L, Li B (2012) Changes in cross-correlations as an indicator for systemic risk. Scientific Reports 2: 888. 23185692 10.1038/srep00888 PMC3506152

[14] Mantegna R, Stanley H (2000) Introduction to Econophysics: Correlations and Complexity in Finance. Cambridge: Cambridge University Press.

[15] Forsberg L, Ghysels E (2007) Why do absolute returns predict volatility so well? Journal of Financial Econometrics 5: 31.

[16] Andersen T, Bollerslev T (1998) Answering the skeptics: Yes, standard volatility models do provide accurate forecasts. International Economic Review 39: 885.

[17] Bedowska-Sójka B, Kliber A (2010) Realized volatility versus garch and stochastic volatility models. the evidence from the wig20 index and the eur/pln foreign exchange market. Statistical Review 57: 105.

[18] Ren F, Gu G, Zhou W (2009) Scaling and memory in return intervals of realized volatility. Physica A 388: 4787.

[19] Roll R (1984) A simple implicit measure of the effective bid-ask spread in an efficient market. Journal of Finance 39: 1127.

[20] Zhou B (1996) High-frequency data and volatility in foreign-exchange rateshigh-frequency data and volatility in foreign-exchange rates. Journal of Business and Economic Statistics 14: 45.

[21] Andersen T, Bollerslev T, Dobrev D (2007) No-arbitrage semi-martingale restrictions for continuous-time volatility models subject to leverage effects, jumps and i.i.d. noise: Theory and testable distributional implications. Journal of Econometrics 138: 125.

[22] Andersen T, Bollerslev T, Frederiksen P, Nielsen MO (2010) Continuous-time models, realized volatilities, and testable distributional implications for daily stock returns. Journal of Applied Econometrics 25: 233.

[23] Takaishi T, Chen T, Zheng Z (2012) Analysis of realized volatility in two trading sessions of the japanese stock market. Prog Theor Phys Suppl 194: 43.

[24] Takaishi T (2012) Finite-sample effects on the standardized returns of the tokyo stock exchange. Procedia: Social and Behavioral Sciences 65: 968.

[25] Shao YH, Gu GF, Jiang ZQ, Zhou W, Sornette D (2012) Comparing the performance of fa, dfa and dma using different synthetic long-range correlated time series. Scientific Reports 2: 835. 23150785 10.1038/srep00835 PMC3495288

[26] Costa M, Goldberger A, Peng C (2002) Multiscale entropy analysis of complex physiologic time series. Phys Rev Lett 89: 068102. 12190613 10.1103/PhysRevLett.89.068102

[27] Andersen T, Bollerslev T, Diebold F, Labys P (2001) The distribution of exchange rate volatility. Journal of the American Statistical Association 96: 42.

[28] Andersen T, Bollerslev T, Diebold F, Ebens H (2001) The distribution of realized stock return volatility. Journal of Financial Economics 61: 43.

[29] Bandi F, Russell J (2008) Microstructure noise, realized variance and optimal sampling. The Review of Economic Studies 75: 339.

[30] Gallos LK, Makse HA, Sigman M (2012) A small world of weak ties provides optimal global integration of self-similar modules in functional brain networks. Proceedings of the National Academy of Sciences 109: 2825. 10.1073/pnas.1106612109 PMC3286928 22308319

[31] Clauset A, Shalizi C, Newman M (2009) Power-law distributions in empirical data. SIAM Rev 51: 661.

